# Effects of Simulated Heat Waves on Cardiovascular Functions in Senile Mice

**DOI:** 10.3390/ijerph110807841

**Published:** 2014-08-06

**Authors:** Xiakun Zhang, Jing Lu, Shuyu Zhang, Chunling Wang, Baojian Wang, Pinwen Guo, Lina Dong

**Affiliations:** 1 School of Atmospheric Science, Nanjing University of Information Science and Technology, 219 Ningliu Road, Nanjing 210044, China; E-Mails: zxk668@126.com (X.Z.); guo@nuist.edu.cn (P.G.); dlina@nuist.edu.cn (L.D.); 2 Computer Science Department, Oklahoma State University, 219 MSCS, Stillwater, OK 74078, USA; E-Mail: lujing19810629@hotmail.com; 3 Key Laboratory of Arid Climatic Change and Reducing Disaster of Gansu Province, Lanzhou Institute of Arid Meteorology, China Meteorological Administration, 2070 Donggang East Road, Lanzhou 730020, China; 4 School of Applied Meteorology, Nanjing University of Information Science and Technology, 219 Ningliu Road, Nanjing 210044, China; E-Mail: wangchunling668@126.com; 5 Lanzhou Central Meteorological Observatory, 2070 Donggang East Road, Lanzhou 730020, China; E-Mail: baojianwang@126.com

**Keywords:** heat wave weather, senile mice, HSP60, SOD, TNF, sICAM-1, atherosclerosis

## Abstract

The mechanism of the effects of simulated heat waves on cardiovascular disease in senile mice was investigated. Heat waves were simulated in a TEM1880 meteorological environment simulation chamber, according to a heat wave that occurred in July 2001 in Nanjing, China. Eighteen senile mice were divided into control, heat wave, and heat wave BH4 groups, respectively. Mice in the heat wave and heat wave BH4 groups were exposed to simulated heat waves in the simulation chamber. The levels of ET-1, NO, HSP60, SOD, TNF, sICAM-1, and HIF-1α in each group of mice were measured after heat wave simulation. Results show that heat waves decreased SOD activity in the myocardial tissue of senile mice, increased NO, HSP60, TNF, sICAM-1, and HIF-1α levels, and slightly decreased ET-1 levels, BH4 can relieve the effects of heat waves on various biological indicators. After a comprehensive analysis of the experiments above, we draw the followings conclusions regarding the influence of heat waves on senile mice: excess HSP60 activated immune cells, and induced endothelial cells and macrophages to secrete large amounts of ICAM-1, TNF-α, and other inflammatory cytokines, it also activated the inflammation response in the body and damaged the coronary endothelial cell structure, which increased the permeability of blood vessel intima and decreased SOD activity in cardiac tissues. The oxidation of lipoproteins in the blood increased, and large amounts of cholesterol were generated. Cholesterol penetrated the intima and deposited on the blood vessel wall, forming atherosclerosis and leading to the occurrence of cardiovascular disease in senile mice. These results maybe are useful for studying the effects of heat waves on elderly humans, which we discussed in the discussion chapter.

## 1. Introduction

Human health is related to the weather. The frequent occurrence of extreme weather in recent years has brought economic losses and endangered human health [1]. Epidemiological studies and statistical studies have demonstrated that heat waves significantly increase the incidence of coronary heart disease (CHD), and many people are admitted to hospitals or die because of heat exposure during heat waves [2]. Kunst *et al.* [3] studied the relationship between extreme weather and mortality from 1979 to 1987, and found that 26% of heat-induced deaths were caused by cardiovascular diseases. Lu *et al.* [4] found that the incidence rate of cardiovascular disease is 35.1% of the total number during hot weather, including the CHD incidence rate of 10.8%. The *China Cardiovascular Report* [5] showed that about 230 million people in China currently suffer from CHD, heart failure, hypertension, and other cardiovascular diseases. Cardiovascular disease is a major disease that threatens the life and health of the elderly. The annual number of deaths caused by cardiovascular disease is nearly 3 million, particularly, cardiovascular events in elderly people showed an increasing trend with the augment of heat wave events due to climate warming [6]. It shows that the heat wave has a significant effect on the prevalence of cardiovascular disease in elderly people. Wang *et al.* [7] studied the effects of heat waves on 8-week-ApoE-/- mice, while we investigated the effects of heat waves on the cardiac functions of senile mice that Wang *et al.* did not refer. In our experiment, senile mice were placed in a meteorological environment simulation chamber and exposed to simulated heat waves according to the heat wave data recorded in Nanjing; the cardiac functions of senile mice were monitored by cardiac biomarker levels, including body temperature and levels of endothelin-1 (ET-1), nitric oxide (NO), heat shock protein 60 (HSP60), superoxide dismutase (SOD), tumor necrosis factor (TNF), soluble intercellular adhesion molecule (sICAM), and hypoxia-inducible factor 1-alpha (HIF-1α); the mechanism of the effects of heat waves on cardiovascular functions in mice was also determined.

Nitric oxide synthase (NOS), a key factor in the production of nitric oxide, plays an important role in its biological function. Many cardiovascular diseases are associated with low NOS activity and NO deficiency. Besides, BH4 belongs to the aromatic amino acid mono-oxygenase system and it has been confirmed that NOS would result in coupling lost between oxygen reduction and L-Arg oxidation in the case of BH4 absence and consequently generate O^2−^ and H_2_O_2_ [8], which illustrate NOS function will show a great reversal in the case of BH4 absence. The increments of the NOS activity demand that BH4, catalytic L-Arg cofactors and so on to promote the formation of NO [9]. Verma *et al.* [10] experimented on isolated rat cardiac and human cardiomyocytes *in vitro*, and found that BH4 inhibits the decline in coronary endothelial functions, and reduces lipid peroxidation and myocardial cell damage.

HSPs are closely related to the development of autoimmune diseases, atherosclerosis and other diseases [11]. HSP60 is an important member of the HSP family. Zhou *et al.* [12] found that the HSP60 levels in the sera of patients with CHD are associated with the condition of coronary artery. Zhang *et al.* [13] proposed that HSP60 expression levels are extremely related with the risk of CHD, and the risk of high expression groups can be several times higher. Li *et al.* [14] showed through experiments in mice that the oral administration of HSP60 induces antigen-specific immune tolerance by amplifying regulatory T cells, thereby inhibiting atherosclerosis. These results showed that HSP60 is related to the formation and development of CHD. Superoxide dismutase (SOD), as a sensitive indicator of heart disease diagnosis, its character changes in the early stage of many cardiovascular and cerebrovascular diseases and is closely related to coronary heart disease. The SOD activity decreased in cardiac tissues, leading to the release of radicals by excessive oxygen in cardiac tissues and an increase in lipid peroxidation. These effects could result in endothelial cell and cardiac dysfunction and myocardial ischemia. Excess reactive oxygen species production directly damages vascular endothelial cells, induces NO inactivation and lipoprotein oxidation in the blood, and causes the deposition of cholesterol in the blood vessel wall, forming atherosclerosis [15,16]. Chen *et al.* [17] pointed out that oxidative damage is an important mechanism in the development of coronary atherosclerosis. TNF is a cytokine with a systemic effect. Its two forms, TNF-α and TNF-β, have similar inflammatory activities [18]. These forms may participate in the inflammatory response and immune response, causing myocardial cell injury or remodeling. Moreover, they are closely related to the degree of myocardial ischemia and occurrence of CHD and other cardiovascular diseases. ICAM is a glycoprotein molecule that regulates mutual recognition, adhesion, and signal transduction of cell-cell or cell-extracellular matrix. ICAM is widely distributed in the body, regulating cell growth, differentiation, and inter-cell interactions, and is involved in inflammatory and immune responses, coagulation and thrombosis, and other physiological and pathological processes [19,20,21]. Studies [20] have shown that blood sICAM-1 is the marker of endothelial cell activation, which reflects the extent of coronary artery inflammation. sICAM-1 is the main inflammatory cytokine in the body. Lin *et al.* [22,23] reported that inflammation starts at adhesion between leukocytes and vascular endothelium. Leukocytes and platelets adhere and aggregate because of sICAM-1. Inflammatory cells adhere to the vascular endothelium and infiltrate into endothelial cells to secrete active substances, leading to vascular smooth muscle cell proliferation and foam cell formation, which result in the formation and development of atherosclerosis and thrombosis; thus, sICAM-1 is closely related to the occurrence and development of atherosclerosis and CHD [22]. Luc *et al.* [24] observed more than 300 patients with CHD for five years, and found that increased plasma levels of sICAM-1 are related to the occurrences of angina, myocardial infarction, and other diseases related deaths. sICAM-1 at 100 ng/mL can increase the risk of coronary events by 30% [25]. TNF and ICAM-1, which may mark the extent of the inflammatory response in the body, are independent risk factors for CHD [26,27,28]. ET-1, a multifunctional bioactive peptide composed of endothelial cell and cardiac cell secretions, is the strongest vasoconstrictor as far as is known [29]. Endothelium-derived NO, which is the main vasodilator in the body, promotes vascular smooth muscle relaxation and vasodilatation. NO and ET-1 are important factors in regulating cardiovascular function. Their changes in the body affect diastolic and systolic pressures of blood vessels, playing an important role in maintaining basic vascular tone and the cardiovascular system [30]. The relaxation of blood vessels directly affects the process of the body heat loss. During heat waves, vasodilation helps heat dissipation through the body surface to avoid the heat influence. HIF-1, a nuclear protein with transcriptional activity that is related to the expression of many genes in hypoxia adaptation and inflammatory processes, promotes the transcription of erythropoietin, vascular endothelial factor, and other target genes [31]. Zhao [32] showed that the HIF-1α levels are associated with the occurrence and the growth of a CHD illness, and HIF may be closely related to the development of ischemic diseases, such as CHD [33].

## 2. Experimental Section

### 2.1. Experimental Equipments and Materials

Test instruments included a TEM1880 meteorological environment simulation chamber provided by Tianjin Sprint Environmental Test Equipment Co., Ltd. (Tianjin, China) that can simulate a combined temperature-humidity-pressure test environment and allow fresh air (oxygen) injection when necessary during the experiments. A TH212 special thermal detector from Hong’ou Science and Technology Co., Ltd. (Beijing, China) were used in this study; a medical centrifuge, electronic balance, ultra-low temperature refrigerator, and microplate reader were also used. Test materials included chloral hydrate, tetrahydrobiopterin (BH4), ET-1 ELISA kit, NO nitrate reductase kit, total SOD hydroxylamine kit, sICAM ELISA kit, HSP60 ELISA kit, and TNF ELISA kit.

### 2.2. Heat Wave Curve

**Figure 1:**
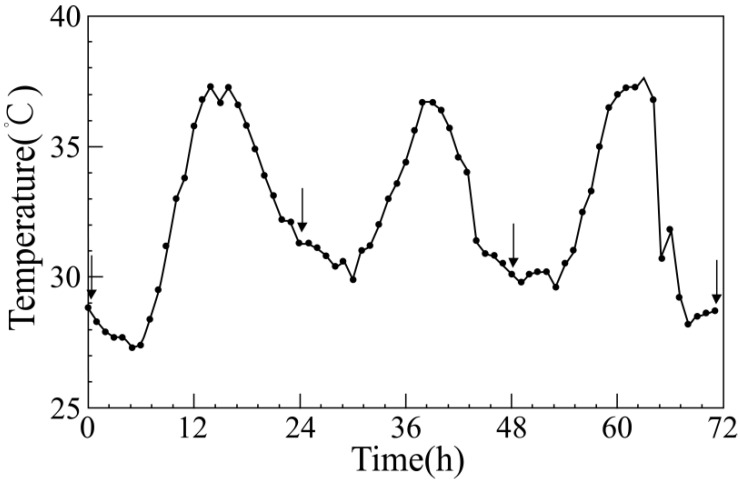
Temperature curve of the simulated heat wave. The *arrows* denote sampling time points when the body temperatures and weights of experimental mice were measured in the three experimental groups.

Hourly meteorological data (temperature, humidity, and precipitation) were collected in Nanjing during the summer seasons (June to August) from 2001 to 2010. According to the China Meteorological Administration criteria, a maximum temperature ≥35 °C on any given day is called high temperature, and three or more consecutive days of high temperature are called a heat wave. A heat wave model was developed based on a heat wave that occurred in July 2001 in Nanjing. The temperature simulation curve is shown in Figure 1. The daily average temperature was 31.8 °C; the average maximum temperature was 36.6; the average duration of heat waves in three days was 5.7 hours. Experiments started from 0 o’clock on 9 July 2001 and ended at 24 o’clock on 11 July. The experimental temperature of the control group was set at 27 °C, which was the average summer temperature in Nanjing from 2001 to 2010.

### 2.3. Feeding and Grouping of Animals

Eighteen 15-week-old specific pathogen-free male mice with an average weight of 30.54 g were fed with adequate animal feed and water for eight weeks in plastic and metal cages under a circadian rhythm of 12 h/12 h, with light supply from 08:00 to 20:00. The cages were stored at 27 °C (average summer temperature in Nanjing) with 45% RH. The animal feed, which contained corn, soybean meal, fish meal, wheat flour, wheat bran, calcium hydrogen phosphate, stone powder, vitamins, trace elements, and amino acids, was purchased from Beijing Ke’ao Xieli Feed Co., Ltd. (Beijing, China). Litters were capsule-shaped corn cobs, which were replaced daily. Mice were caught from cage for several times per day to reduce the additional effects caused by the capture of mice during the experimental process. The 18 senile mice were randomly divided into three groups, namely, the control group (n = 6), heat wave group (n = 6), and heat wave BH4 group (n = 6).

The animal protocols used in this work were evaluated and approved by the Animal Use and Ethic Committee of the Institute of Arid Meteorology, China Meteorological Administration (Protocol No. 2014_1). They are in accordance with *Guidance Suggestions for the Care and Use of Laboratory Animals* (issued by the Ministry of Science and Technology of the People’s Republic of China, document No. 2006_398) and the *Regulations for Laboratory Animal Management* (revised by Decree of the State Council of the People’s Republic of China, No. 638).

### 2.4. Experimental Scheme

The heat wave BH4 group received gavage with 10 mg/kg BH4 one day before the adaptation period. The control and heat wave groups received gavage with equal amounts of standard saline. After the control period, mice in the heat wave and heat wave BH4 groups were exposed to complete heat wave treatment in the meteorological environment simulation chamber, as shown in Figure 1. Mice in the control group were kept in the same environment, except for heat wave treatment. Each group of mice was allowed to eat and drink freely.

### 2.5. Monitoring and Collection of Indicators

The heat wave treatment lasted for 72 h. The conditions of mice were observed during the experiment, and the body weights and temperatures in each group were measured daily at the specific time points illustrated in Figure 1. Mice of the heat wave BH4 group were fed with the drug through gavage. In humans, treatment with BH4 can also improve cutaneous vascular function in aging [34,35]. The mice were removed from the chamber after heat wave simulation, and anaesthetized (7% chloral hydrate, 0.3 mL/100 g). Blood samples (approximately 1 mL each) were collected by decollating into centrifuge tubes. Plasma samples were separated by centrifugation at 3,000 rpm for 15 min, and stored in refrigerators at −20 °C until analysis. Heart tissues were homogenized in 0.9% saline and centrifuged at 3,000 rpm for 15 min. The supernatant was collected and stored in refrigerators at −20 °C until analysis.

The frozen plasma was thawed at 37 °C before measurement. ET-1, sICAM-1, and TNF levels in plasma were measured using ELISA assay kits and a microplate reader. Plasma NO level was determined by the nitrate reductase method. Frozen myocardial tissue fluids of mice were similarly reconstituted. SOD activity levels in cardiac tissues were determined by the hydroxylamine method. The sICAM-1 and HSP60 levels in the heart were measured using ELISA assay kits.

### 2.6. Statistical Analysis

Data were analyzed using SPSS19.0, and shown as mean ± standard error. Differences between treatment groups were interpreted by one-way ANOVA with the least square difference *post hoc* multiple comparisons. Differences of *p* < 0.05 were considered statistically significant.

## 3. Results and Discussion

### 3.1. Body Weight and Rectal Temperature

**Figure 2:**
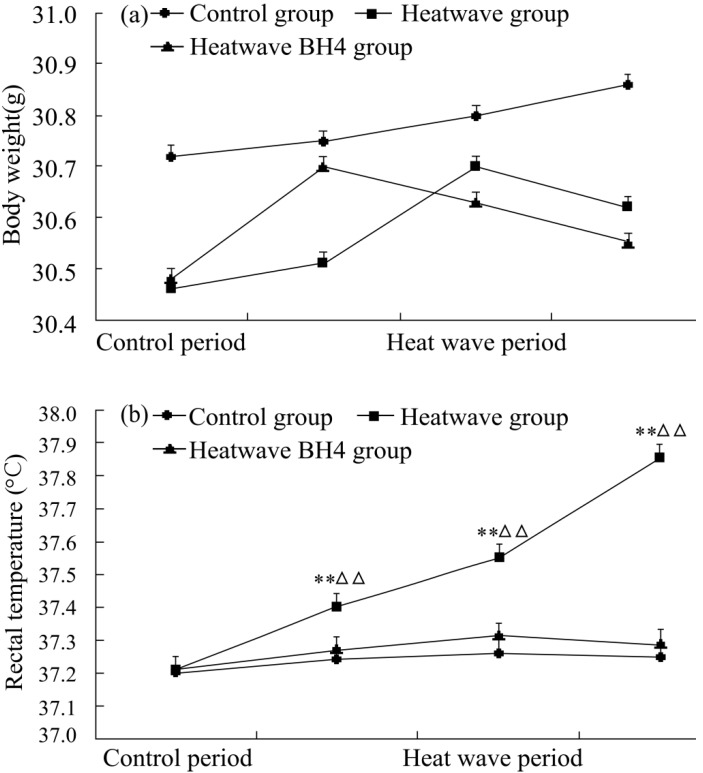
Body weights (**a**) and rectal temperatures (**b**) of senile mice in the control, heat wave, and heat wave BH4 groups (heat wave exposure for 3 d). ** *p* < 0.01 *vs*. control group; △△ *p* < 0.01 *vs*. heat wave BH4 group at the same time of measurement.

The body weights and rectal temperatures of senile mice were monitored daily during the 3 d of the experiment. Figure 2 shows that the body weights in all three groups slightly increased by the end of the experiment (*p* > 0.05). Mice in each group showed increased rectal temperature with the progression of the heat process between the start and the end of the experiment. Rectal temperature significantly increased by 0.65 °C in the heat wave group, whereas that of the heat wave BH4 and control groups slightly increased by 0.07 °C and 0.05 °C, respectively. Rectal temperature increases in the heat wave group were significantly higher than those in the control group (*p* < 0.01), but the rectal temperature differences between the heat wave BH4 and control groups were not statistically significant (*p* > 0.05).

### 3.2. Analysis of the Changes in HSP60

**Figure 3:**
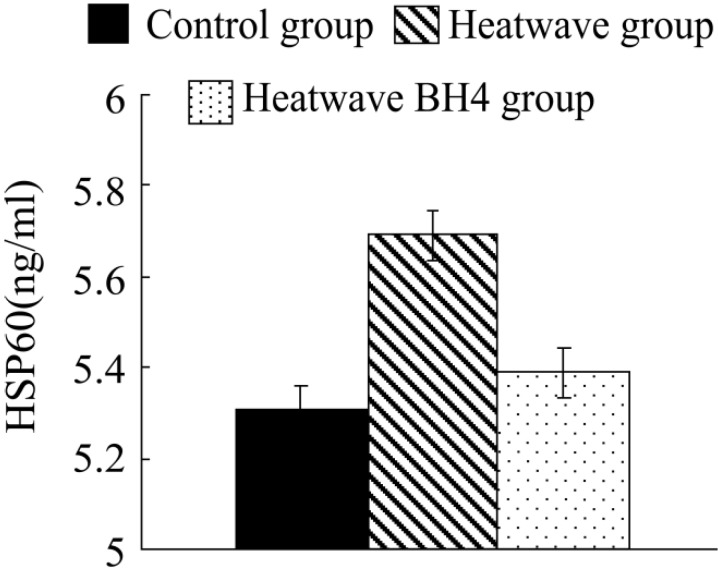
HSP60 levels in senile mice at the end of the experiment.

The HSP60 levels in senile mice cardiac tissues of different groups in heat wave experiments (Figure 3) illustrated: compared with the control group, these HSP60 values of the two groups have different increasing in different degrees by 0.382 and 0.081 ng/mL respectively, while that of the wave BH4 group were 0.381 ng/mL lower than those of the heat wave group, with no significant difference. In conclusion, the heat wave could induce the increments of HSP60 in myocardial tissues of senile mice.

### 3.3. Analysis of TNF and sICAM-1

Table 1 shows the TNF and sICAM-1levels in different groups of senile mice at the end of the experiments. The TNF contents of each group were analyzed. The TNF level in the heat wave group of senile mice was 0.22 pg/mL higher than that in the control group. The effects of BH4 mitigated the changes in TNF levels of mice in the BH4 group *in vivo*. Compared with the control group, the TNF level in the BH4 group slightly increased by 0.06 pg/mL, but with no significant difference between groups (*p* > 0.05). Heat wave increased the TNF levels of the body, but the increase was minimal. Compared with the control group, the sICAM-1 levels of the heat wave group significantly increased by 68.23 pg/mL (*p* < 0.01). The sICAM-1 levels of the heat wave group increased by 49.61 pg/mL than those of the heat wave BH4 group, with a statistically significant difference between groups (*p* < 0.01). The sICAM-1 levels of the heat wave BH4 group increased by 18.62 pg/mL compared with those of the control group; the increase was minimal but significant (*p* < 0.01). These results show that heat waves significantly increased the secretion of sICAM-1 in senile mice. BH4 via gavage alleviated the effect of heat waves on the secretion of sICAM-1 and TNF in senile mice.

**Table 1:** TNF and sICAM-1 levels in senile mice at the end of the experiments.

		Group(s)	TNF (pg/mL)	sICAM-1 (pg/mL)
**Mean ± SE**		Control	7.18 ± 0.804	121.19 ± 6.244
		Heat wave	7.40 ± 0.442	189.42 ± 8.246
		Heat wave BH4	7.24 ± 0.923	139.81 ± 2.651
*p* **value**	**of ANOVA among**	all three groups	0.349	0.000
	**of** *post hoc* **test between**	Control & heat wave	0.286	0.000
		Control & heat wave BH4	0.469	0.003
		Heat wave & heat wave BH4	0.394	0.000

### 3.4. Analysis of Changes in SOD Activity

**Figure 4:**
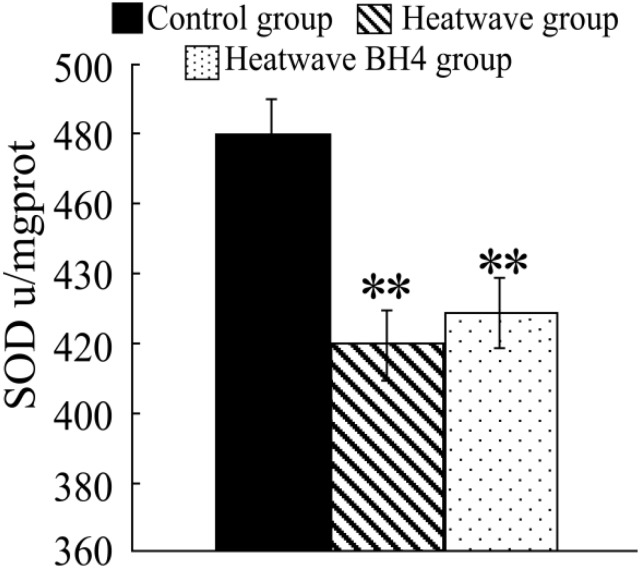
SOD activity in senile mice at the end of the experiment. ** *p* < 0.01 *vs.* control group.

Figure 4 shows that the SOD activity in the heat wave group significantly decreased compared with that in the control group (*p* < 0.05), with a decline of 61.07 u/mg pro. The SOD activity in the heat wave BH4 group also decreased, with a decline of 51.71 u/mg pro, and the differences were statistically significant (*p* < 0.05). The SOD activity in the BH4 group was 9.36 u/mg pro more than that in the heat wave group with no significant difference (*p* > 0.05). The SOD activity of senile mice exposed to heat wave for 3 d decreased, whereas BH4 alleviated the effect of heat wave on the decline in SOD activity in senile mice.

The experimental results show that heat waves decreased SOD activity in senile mice and caused excess cardiac oxygen free radicals, which increased lipoprotein oxidation in the blood and accelerated cholesterol deposition in the vessel walls, forming atherosclerosis and promoting the occurrence and development of cardiovascular diseases.

### 3.5. Levels of ET-1, NO, and ET-1/NO

Table 2 shows the ET-1 levels in senile mice of each group declined slightly during the experiment. Compared with the control group, the ET-1 level in the heat wave group decreased by 3.47 μmol/L, and that in the heat wave BH4 group decreased by 0.19 μmol/L with no significant difference (*p* > 0.05).

**Table 2:** ET-1 levels, NO levels, and NO/ET-1 ratios in senile mice at the end of the experiments.

		Group(s)	ET-1 (ng/L)	NO (μmol/L)	NO/ET-1 (%)
**Mean ± SE**		Control	164.38 ± 10.53	47.39 ± 6.77	28.62 ± 2.21
		Heat wave	160.91 ± 7.39	62.06 ± 4.87	38.49 ± 1.84
		Heat wave BH4	164.19 ± 16.21	90.47 ± 9.15	55.19 ± 1.63
*p* **value**	**of ANOVA among**	all three groups	0.905	0.000	0.000
	**of** *post hoc* **test between**	Control & heat wave	0.321	0.025	0.001
		Control & heat wave BH4	0.486	0.000	0.000
		Heat wave & heat wave BH4	0.368	0.000	0.000

Table 2 also shows the NO levels in senile mice at the end of the experiment. The NO levels in the heat wave and heat wave BH4 groups were both significantly higher than that in the control group (*p* < 0.01). Compared with the control group, the NO level in the heat wave group increased by 14.67 μmol/L, and that in the heat wave BH4 group increased by 43.08 μmol/L. The NO level in the heat wave BH4 group was significantly higher than that in the heat wave group (*p* < 0.05).

The NO/ET-1 ratios in senile mice at the end of the experiment are shown in Table 1. Trends in the NO/ET-1 ratios largely resembled those of NO levels. The NO/ET-1 ratios in both the heat wave and heat wave BH4 groups were significantly higher than that in the control group (*p* < 0.01). The NO/ET-1 ratio in the heat wave group rose by 9.87%, and that of the heat wave BH4 group rose by 26.57%. The NO/ET-1 ratio of the heat wave BH4 group increased by 16.7% compared with that of the heat wave group. Significant differences between the two groups were observed (*p* < 0.01).

The results show that the NO levels in the heat wave and heat wave BH4 groups significantly increased after heat exposure. The administration of BH4 to senile mice resulted in a more pronounced increase in NO levels. A comparative analysis among the rectal temperature measurements in senile mice in each group showed that the rectal temperature of heat wave mice exhibited a more pronounced consecutive daily increase during 3 d of heat exposure, whereas smaller increases were observed in the control and heat wave BH4 groups. The NO contents in mice of each group were sorted from highest to lowest, its value in the heat wave BH4 group was 43.08 μmol/L higher than that of the control group, and 28.41 μmol/L higher than that of the heat wave group after heat exposure. The rectal temperature measurements of each group were compared with those of the control group. The results show that the increase in NO was significantly lower in the heat wave group than that in the control group. Moreover, rectal temperature was significantly higher in the heat wave group than that in the control group. By contrast, the increase in rectal temperature was smaller in the heat wave BH4 group, which had the highest NO content, than that in the control group.

The effect of heat wave stimulation on the ET-1 levels in mice was very small, and the ET-1 levels only showed a minimal decline. Heat waves significantly increased the NO levels in senile mice, making the NO/ET-1 balance favor vasodilation, enhancing body heat dissipation, and promoting the decline in temperature. As the heat wave progressed, NO released by the senile mouse end othelium was insufficient to mitigate the effects of the heat wave. Thus, the mice body temperature notably increased as the heat wave progressed. The experimental results show that BH4, as a NOS synthase, promoted NO release in senile mice *in vivo*, enhancing the cooling efficiency and reducing heat hazards to senile mice.

### 3.6. Analysis of HIF-1α Levels

This experiment was performed by heat stimulation on mice to determine the HIF content changes in the animal body during heat waves.

**Figure 5:**
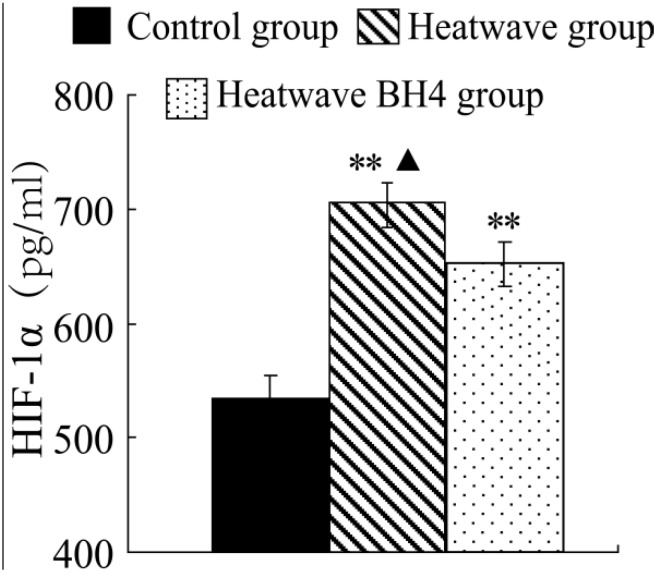
HIF-1α levels in senile mice at the end of the experiment. ** *p* < 0.01 *vs*. control group, △ *p* < 0.05 *vs*. heat wave BH4 group.

Figure 5 shows that the HIF-1α level of elderly mice in the heat group significantly increased by 169.68 pg/mL at the end of heat exposure compared with that in the control group (*p* < 0.01). The HIF-1α expression levels in the heart homogenates of mice in the heat wave BH4 group were lower at 51.63 pg/mL than those of the heat wave group (*p* < 0.05), but higher at 118.05 pg/mL than those of the control group (*p* < 0.01). Heat waves were observed to induce the increase in HIF-1α expression in senile mice.

## 4. Discussion and Conclusions

An actual heat wave in Nanjing was simulated using a meteorological environment simulation chamber. The effect of heat waves on senile mice and function of BH4 in the heat stress of the body were preliminarily analyzed by determining body weight, rectal temperature, and levels of ET-1, NO, HSP60, SOD, TNF, sICAM-1, and HIF-1α in senile mice before and after heat wave simulation. Changes in each index in mice treated with BH4 were also analyzed.

### 4.1. Discussion

SOD is an important indicator that maintains oxidative balance and vascular endothelial function in the body [7,16,36]. This experiment showed that the heat stimulation can significantly decrease the SOD activity in cardiac tissues of senile mice. BH4 could mitigate the effect of heat waves on SOD activity. The exogenous balance of BH4 may reduce the harm of heat waves on cardiovascular of senile mice.

This study showed heat waves slightly decreased the ET-1 levels and significantly increased the NO levels in senile mice, making the NO/ET-1 balance favor vasodilation, enhancing body heat dissipation, and promoting the decline in temperature [26]; it also showed that BH4, as a NOS synthase, can promote the release of NO in senile mice *in vivo* and reduce the effect of heat waves on cardiovascular disease. Previous studies [37,38] indicated that excessive HSP60 in myocardium can activate immune cells, induce endothelial cells and macrophages to secrete a large amount of ICAM-1, TNF-α, and other inflammatory cytokines. Then the immune response is activated, making HSP60 locate on the surface of macrophages and then smooth muscle cells bind its own antibodies that results in the generation of the antibody complexes that can damage endodermis, increase inflammatory cell adhesion, lipidosis, and atherosclerosis finally. This experiment showed that high temperature can elevate the HSP60 expression levels in myocardium of senile mice, which then can accelerate atherosclerosis, and result in the occurring and development of CHD; the BH4 can decrease HSP60 expression levels in myocardium of senile mice, thereby it can relieve the effect of heat waves on coronary artery of senile mice; heat wave exposure induces the increment of the secretion of cytokines TNF and sICAM-1 in senile mice, while for the senile mice group that added BH4, they are less subjected to the heat waves with obvious lower TNF and sICAM-1compared with the heat waves group. After analyzing the gained results comprehensively, Leukocytes and platelets can adhere and aggregate through the mediation of sICAM-1 and TNF, inflammatory cells can adhere to the vascular endothelium and infiltrate endothelial cells to secrete active substances, leading to the proliferation of vascular smooth muscle cell, which then forms the foam cell that accelerates the formation and development of atherosclerosis and even thrombosis in serious cases [39]. Therefore, heat waves can lead to the occurrence and development of cardiovascular diseases.

BH4 can relieve the increased expression of inflammatory factors caused by heat wave stimulation, thereby protecting the cardiovascular system. This study showed that Heat waves can induce the increment of HIF-1α expression in senile mice. Cramer *et al*. [40] demonstrated through *in vitro* bone marrow cell experiments that HIF-1α is the key factor in the adhesion and migration of invasion, and HIF-1α deletion may inhibit inflammation. Sluimer *et al*. [33] demonstrated that carotid atherosclerotic plaques increase the occurrence of hypoxia and HIF-1α expression, leading to the further development of atherosclerosis. Thus, heat stimulation can increase the risk of cardiovascular disease by inducing the expression of HIF-1α in myocardial tissues BH4 while supplementation can alleviate the effect caused by thermal stimulation and provides protective effects to the body.

### 4.2. Conclusions

1. Heat waves decreased SOD activity in the myocardial tissue of senile mice, increased NO, HSP60, TNF, sICAM-1, and HIF-1α levels, and slightly decreased ET-1 levels, increased body weight and body temperature, BH4 can relieve effects of heat waves on various biological indicators.

2. Based on the results of the experiments and discussion, we can consider the possible mechanism of heat wave weather effects on cardiovascular disease in senile mice is as follows. Heat waves slightly decrease the ET-1 levels and significantly increase the NO levels in senile mice, making the NO/ET-1 balance favor vasodilation, enhancing body heat dissipation, and promoting a decline in temperature. NO released by the senile mouse endothelium as the heat wave progresses is insufficient in mitigating the effects of the heat wave, thereby increasing the mice body temperature as the heat wave progresses. Heat waves induce the increase in HSP60 in myocardial tissues of senile mice. Excessive HSP60 activates immune cells, and induces endothelial cells and macrophages to secrete large amounts of ICAM-1, TNF-α, and other inflammatory cytokines. Increased sICAM-1 and TNF-α in the plasma of the body initially activates inflammation. Increased HIF-1α expression further exacerbates the inflammatory response. Leukocyte and platelet adhesion and aggregation are mediated by sICAM-1. Inflammatory cells can adhere to the vascular endothelium and infiltrate endothelial cells to secrete active substances. These substances lead to vascular smooth muscle cell proliferation and foam cell formation, resulting in the formation and development of atherosclerosis and thrombosis. Heat waves can also decrease the SOD activity in heart tissues, and cause excessive cardiac oxygen free radicals. Lipoprotein oxidation in the blood increases and deposition of cholesterol in the vessel wall accelerates, further promoting atherosclerosis and increasing the risks of cardiovascular disease.

## Supplementary Files

Supplementary File 1
