# Supplementary material for: Effects of Simulated Heat Waves on Cardiovascular Functions in Senile Mice
Source: Int J Environ Res Public Health. 2014 Aug 6;11(8):7841–55. doi: 10.3390/ijerph110807841 (PMC4143836; doi:10.3390/ijerph110807841)
Supplement: Supplementary File 1 [file ijerph-11-07841-s001.pdf]

Supplementary Material

Role of Metabolic Genes in Blood Arsenic Concentrations of Jamaican Children with and without Autism Spectrum Disorder

---

Detailed description of General Linear Models used to obtain the results reported in Table 3 (based on 100 matched pairs)

General Linear Models that assessed unadjusted effects of various exposure variables in blood arsenic concentrations of children with and without ASD can be represented by the following linear regression equations:

Models: $E(Y_i \mid X_{1i}, X_{2i}, X_{12ij}) = \beta_0 + \beta_1 X_{1i} + \beta_{2k} X_{2ki} + \sum_{j=1}^{99} \beta_{3j} X_{3ij}$; *k=1, 2,..., 27 that represent various exposure variables*

$$i = 1, 2, \dots, 200\ (number\ of\ children);\ j = 1, 2, \dots, 99 (number\ of\ dummy\ variables\ representing\ the\ 100\ matched\ pairs);$$

$$Y_i = ln\ of\ blood\ arsenic\ concentrations\ of\ the\ i^{th}\ child;$$

$$X_{1i} = case\ status\ of\ the\ i^{th}\ child\ (ASD\ case = 1;\ TD\ control = 0);$$

$$X_{21i} = car\ ownership\ associated\ with\ family\ of\ the\ i^{th}\ child\ (No\ cars = 0; otherwise = 1);$$

$$X_{22i} = maternal\ age\ at\ child's\ birth\ associated\ with\ the\ i^{th}\ child\ (\text{"}maternal\ age \geq 35\ years\text{"} = 1;\ otherwise = 0);$$

$$X_{23i} = parental\ levels\ of\ education\ associated\ with\ the\ i^{th}\ child\ (both\ parents\ had\ up\ to\ high\ school = 0; otherwise = 1);$$

$$X_{24i} = source\ of\ drinking\ water\ associated\ with\ the\ i^{th}\ child\ (piped\ water = 1;\ otherwise = 0);$$

$$X_{25i} = consumption\ of\ \text{“}yam, sweet\ potato, or\ dasheen\text{”}\ associated\ with\ the\ i^{th}\ child\ (No\ consumption = 0;\ otherwise = 1);$$

$$X_{26i} = consumption\ of\ \text{“}carrot\ or\ pumpkin\text{”}\ associated\ with\ the\ i^{th}\ child\ (No\ consumption = 0;\ otherwise = 1);$$

$$X_{27i} = consumption\ of\ \text{“}lettuce\text{”}\ associated\ with\ the\ i^{th}\ child\ (No\ consumption = 0;\ otherwise = 1);$$

$$X_{28i} = consumption\ of\ \text{“}callaloo, broccoli, or\ pak\ choi\text{”}\ associated\ with\ the\ i^{th}\ child\ (No\ consumption = 0;\ otherwise = 1);$$

$$X_{29i} = consumption\ of\ \text{“}cabbage\text{”}\ associated\ with\ the\ i^{th}\ child\ (No\ consumption = 0;\ otherwise = 1);$$

$$X_{210i} = consumption\ of\ \text{“}string\ beans\text{”}\ associated\ with\ the\ i^{th}\ child\ (No\ consumption = 0;\ otherwise = 1);$$

$$X_{211i} = consumption\ of\ \text{“}tomatoes\text{”}\ associated\ with\ the\ i^{th}\ child\ (No\ consumption = 0;\ otherwise = 1);$$

$$X_{212i} = consumption\ of\ \text{“}ackee\text{”}\ associated\ with\ the\ i^{th}\ child\ (No\ consumption = 0;\ otherwise = 1);$$

$$X_{213i} = consumption\ of\ \text{“}avocado\text{”}\ associated\ with\ the\ i^{th}\ child\ (No\ consumption = 0;\ otherwise = 1);$$

$$X_{214i} = high\ seafood\ consumption\ associated\ with\ the\ i^{th}\ child\ (more\ than\ 6\ seafood\ meals\ per\ week = 1;\ otherwise = 0);$$

$$X_{215i} = frequency\ of\ seafood\ consumption\ associated\ with\ the\ i^{th}\ child = number\ of\ seafood\ meals\ consumed\ in\ a\ week;$$

$$X_{216i} = consumption\ of\ \text{“}salt\ water\ fish\text{”}\ associated\ with\ the\ i^{th}\ child\ (No\ consumption = 0;\ otherwise = 1);$$

$$X_{217i} = consumption\ of\ \text{“}fresh\ water\ fish\text{”}\ associated\ with\ the\ i^{th}\ child\ (No\ consumption = 0;\ otherwise = 1);$$

$$X_{218i} = consumption\ of\ \text{“}sardine, mackerel\text{”}\ associated\ with\ the\ i^{th}\ child\ (No\ consumption = 0;\ otherwise = 1);$$

$$X_{219i} = consumption\ of\ \text{“}tuna\text{”}\ associated\ with\ the\ i^{th}\ child\ (No\ consumption = 0;\ otherwise = 1);$$

$$X_{220i} = consumption\ of\ \text{“}salted\ fish\ \text{”}\ associated\ with\ the\ i^{th}\ child\ (No\ consumption = 0;\ otherwise = 1);$$

$$X_{221i} = consumption\ of\ \text{“}shellfish\text{”}\ associated\ with\ the\ i^{th}\ child\ (No\ consumption = 0;\ otherwise = 1);$$

$$X_{222i} = consumption\ of\ \text{“}shrimp\text{”}\ associated\ with\ the\ i^{th}\ child\ (No\ consumption = 0;\ otherwise = 1);$$

$$X_{223i} = GSTM1\ genotype\ associated\ with\ the\ i^{th}\ child\ (\text{"}DD\text{"} = 0; \text{"}I/I\text{"}\ or\ \text{"}I/D\text{"} = 1);$$

$$X_{224i} = GSTT1\ genotype\ associated\ with\ the\ i^{th}\ child\ (\text{"}DD\text{"} = 0; \text{"}I/I\text{"}\ or\ \text{"}I/D\text{"} = 1);$$

$$X_{225i} = \text{"}GSTP1(Ile/Ile)\ genotype\text{"}\ associated\ with\ the\ i^{th}\ child\ (GSTP1(Ile/Ile)\ genotype = 1;\ otherwise = 0);$$

$$X_{226i} = \text{“}GSTP1(Val/Val)\ genotype\text{”}\ associated\ with\ the\ i^{th}\ child\ (GSTP1(Val/Val)\ genotype = 1;\ otherwise = 0);$$

$$X_{227i} = \text{“}GSTP1(Ile/Val)\ genotype\text{”}\ associated\ with\ the\ i^{th}\ child\ (GSTP1(Ile/Val)\ genotype = 1;\ otherwise = 0);$$

$$X_{3ij} = \begin{cases} 1, if\ the\ i^{th}\ child\ in\ the\ j^{th}\ matched\ pair\ is\ an\ ASD\ case \\ 0, if\ the\ i^{th}\ child\ in\ the\ j^{th}\ matched\ pair\ is\ a\ TD\ control \end{cases} ;$$

$$E(Y_i | X_{1i}, X_{2ki}, \dots, , X_{3ij})\ stands\ for\ mean\ ln\ blood\ arsenic\ concentrations\ given\ the\ X_{1i}, X_{2ki}, X_{3ij};$$

$$\beta_0, \beta_1, \beta_{2k}, \beta_{3j}\ are\ regression\ coefficients\ associated\ with\ the\ General\ Linear\ Models.$$

Detailed description of additive General Linear Models that are used to obtain the results reported in Table 4 (based on 65 matched pairs)

The univariable and final multivariable additive General Linear Models used that assessed possible association between blood arsenic concentrations and ASD status can be represented by the following linear regression equations:

For the univariable model: $E(Y_i | X_{1i}, X_{12ij}) = \beta_{10} + \beta_{11} X_{1i} + \sum_{j=1}^{64} \beta_{112j} X_{12ij}$;

For the final multivariable model: $E(Y_i | X_{1i}, X_{2i}, \ldots, X_{11i}, X_{12ij}) = \beta_{20} + \beta_{21} X_{1i} + \beta_{22} X_{2i} + \cdots + \beta_{211} X_{11i} + \sum_{j=1}^{64} \beta_{212j} X_{12ij}$;

$$i = 1, 2, \ldots, 130 \text{ (number of children)}; \; j = 1, 2, \ldots, 64 \text{(number of dummy variables representing the 65 matched pairs)};$$

$$Y_i = \ln \text{ of blood arsenic concentrations of the } i^{th} \text{ child};$$

$$X_{1i} = \text{case status of the } i^{th} \text{ child (ASD case} = 1; \text{ TD control} = 0);$$

$$X_{2i} = \text{car ownership associated with family of the } i^{th} \text{ child (No cars} = 0; \text{otherwise} = 1);$$

$$X_{3i} = \text{maternal age at child's birth associated with the } i^{th} \text{ child ("maternal age} \geq 35 \text{ years"} = 1; \text{ otherwise} = 0);$$

$$X_{4i} = \text{Parental levels of education associated with the } i^{th} \text{ child (both parents had up to high school} = 0; \text{otherwise} = 1);$$

$$X_{5i} = \text{source of drinking water associated with the } i^{th} \text{ child (piped water} = 1, \text{ otherwise} = 0);$$

$$X_{6i} = \text{consumption of "yam, sweet potato, or dasheen" associated with the } i^{th} \text{ child (No consumption} = 0; \text{ otherwise} = 1);$$

$$X_{7i} = \text{consumption of "carrot or pumpkin" associated with the } i^{th} \text{ child (No consumption} = 0; \text{ otherwise} = 1);$$

$$X_{8i} = \text{consumption of "callaloo, broccoli, or pak choi" associated with the } i^{th} \text{ child (No consumption} = 0; \text{ otherwise} = 1);$$

$$X_{9i} = \text{consumption of "cabbage" associated with the } i^{th} \text{ child (No consumption} = 0; \text{ otherwise} = 1);$$

$$X_{10i} = \text{consumption of "avocado" associated with the } i^{th} \text{ child (No consumption} = 0; \text{ otherwise} = 1);$$

$$X_{11i} = \text{frequency of seafood consumption associated with the } i^{th} \text{ child} = \text{number of seafood meals consumed in a week};$$

$$X_{12ij} = \begin{cases} 1, \text{if the } i^{th} \text{ child in the } j^{th} \text{ matched pair is an ASD case} \\ 0, \text{if the } i^{th} \text{ child in the } j^{th} \text{ matched pair is a TD control} \end{cases} ;$$

$$E(Y_{1i} | X_{1i}, X_{12ij}) \text{ stands for mean ln blood arsenic concentrations given } X_{1i}, X_{12ij};$$

$$E(Y_{2i} | X_{1i}, X_{2i}, \ldots, X_{11i}, X_{12ij}) \text{ stands for mean ln blood arsenic concentrations given } X_{1i}, X_{2i}, \ldots, X_{11i}, X_{12ij};$$

$$\beta_{10}, \beta_{11}, \beta_{112j} \text{ are regression coefficients associated with the univaribale model};$$

$$\beta_{20}, \beta_{21}, \ldots, \beta_{211}, \beta_{212j} \text{ are regression coefficients associated with the final multivaribale model.}$$

Detailed description of additive General Linear Models used to obtain the results reported in Table 4 (based on 100 matched pairs)

The univariable and final multivariable additive General Linear Models used that assessed possible association between blood arsenic concentrations and ASD status can be represented by the following linear regression equations:

For the univariable model: $E(Y_i|\, X_{1i}, X_{12ij}) = \beta_{10} + \beta_{11} X_{1i} + \sum_{j=1}^{99} \beta_{112j} X_{12ij}$;

For the final multivariable model: $E(Y_i|\, X_{1i}, X_{2i}, \ldots, X_{11i}, X_{12ij}) = \beta_{20} + \beta_{21} X_{1i} + \beta_{22} X_{2i} + \cdots + \beta_{211} X_{11i} + \sum_{j=1}^{99} \beta_{212j} X_{12ij}$;

$$i = 1, 2, \ldots, 200\ (number\ of\ children);\ j = 1, 2, \ldots, 99 (number\ of\ dummy\ variables\ representing\ the\ 100\ matched\ pairs);$$

$$Y_i = ln\ of\ blood\ arsenic\ concentrations\ of\ the\ i^{th}\ child;$$

$$X_{1i} = case\ status\ of\ the\ i^{th}\ child\ (ASD\ case = 1;\ TD\ control = 0);$$

$$X_{2i} = car\ ownership\ associated\ with\ family\ of\ the\ i^{th}\ child\ (\ No\ cars = 0; otherwise = 1);$$

$$X_{3i} = maternal\ age\ at\ child\text{'}s\ birth\ associated\ with\ the\ i^{th}\ child\ (\text{"}maternal\ age \geq 35\ years\text{"} = 1;\ otherwise = 0);$$

$$X_{4i} = Parental\ levels\ of\ education\ associated\ with\ the\ i^{th}\ child\ \ (both\ parents\ had\ up\ to\ high\ school = 0; otherwise = 1);$$

$$X_{5i} = source\ of\ drinking\ water\ associated\ with\ the\ i^{th}\ child\ (piped\ water = 1,\ otherwise = 0);$$

$$X_{6i} = consumption\ of\ \text{“}yam, sweet\ potato, or\ \ dasheen\text{”}\ associated\ with\ the\ i^{th}\ child\ (No\ consumption = 0;\ otherwise = 1);$$

$$X_{7i} = consumption\ of\ \text{“}carrot\ or\ pumpkin\text{”}\ associated\ with\ the\ i^{th}\ child\ (No\ consumption = 0;\ otherwise = 1);$$

$$X_{8i} = consumption\ of\ \ \text{“}callaloo, broccoli, or\ \ pak\ choi\text{”}\ associated\ with\ the\ i^{th}\ child\ (No\ consumption = 0;\ otherwise = 1);$$

$$X_{9i} = consumption\ of\ \text{“}cabbage\text{”}\ associated\ with\ the\ i^{th}\ child\ (No\ consumption = 0;\ otherwise = 1);$$

$$X_{10i} = consumption\ of\ \text{“}avocado\text{”}\ associated\ with\ the\ i^{th}\ child\ (No\ consumption = 0;\ otherwise = 1);$$

$$X_{11i} = frequency\ of\ seafood\ consumption\ associated\ with\ the\ i^{th}\ child = number\ of\ seafood\ meals\ consumed\ in\ a\ week;$$

$$X_{12ij} = \begin{cases} 1, if\ the\ i^{th}\ child\ in\ the\ j^{th}\ matched\ pair\ is\ an\ ASD\ case \\ 0, if\ the\ i^{th}\ child\ in\ the\ j^{th}\ matched\ pair\ is\ a\ TD\ control \end{cases} ;$$

$$E(Y_{1i}|\, X_{1i}, X_{12ij})\ stands\ for\ mean\ ln\ of\ blood\ arsenic\ concentrations\ given\ X_{1i}, X_{12ij};$$

$$E(Y_{2i}|X_{1i}, X_{2i}, \ldots,, X_{12ij})\ stands\ for\ mean\ ln\ of\ blood\ arsenic\ concentrations\ given\ X_{1i}, X_{2i}, \ldots, X_{11i}, X_{12ij};$$

$$\beta_{10}, \beta_{11}, \beta_{112j}\ are\ regression\ coefficients\ associated\ with\ the\ univaribale\ model;$$

$$\beta_{20}, \beta_{21}, \ldots, \beta_{211}, \beta_{12j}\ are\ regression\ coefficients\ associated\ with\ the\ final\ multivaribale\ model.$$

Detailed description of General Linear Models used to obtain the results reported in Table 5 (based on 100 matched pairs)

General Linear Models that assessed unadjusted and adjusted effects of *GSTP1* in blood arsenic concentrations of children with and without ASD can be represented by the following linear regression equations:

For the unadjusted model: $E(Y_i \mid X_{1i}, X_{2i}, X_{12ij}) = \beta_{10} + \beta_{11} X_{1i} + \beta_{12} X_{2i} + \beta_{13} X_{1i} * X_{2i} + \sum_{j=1}^{99} \beta_{113j} X_{13ij};$

For the final adjusted model: $E(Y_i \mid X_{1i}, X_{2i}, \ldots, X_{11i}, X_{12ij}) = \beta_{20} + \beta_{21} X_{1i} + \beta_{22} X_{2i} + \cdots + \beta_{212} X_{12i} + \beta_{13} X_{1i} * X_{2i} + \sum_{j=1}^{99} \beta_{213j} X_{13ij};$

$$i = 1, 2, \ldots, 200\ (number\ of\ children);\ j = 1, 2, \ldots, 99 (number\ of\ dummy\ variables\ representing\ the\ 99\ matched\ pairs);$$

$$Y_i = ln\ of\ blood\ arsenic\ concentrations\ of\ the\ i^{th}\ child;$$

$$X_{1i} = case\ status\ of\ the\ i^{th}\ child\ (ASD\ case = 1;\ TD\ control = 0);$$

$$X_{2i} = GSTP1\ genotype\ associated\ with\ the\ i^{th}\ child\ (GSTP1\ is\ categorized\ into\ 2\ levels);$$

$$X_{3i} = car\ ownership\ associated\ with\ family\ of\ the\ i^{th}\ child\ (No\ cars = 0; otherwise = 1);$$

$$X_{4i} = maternal\ age\ at\ child's\ birth\ associated\ with\ the\ i^{th}\ child\ (\text{"}maternal\ age \geq 35\ years\text{"} = 1;\ otherwise = 0);$$

$$X_{5i} = Parental\ levels\ of\ education\ associated\ with\ the\ i^{th}\ child\ (both\ parent\ up\ to\ high\ schhol = 0; otherwise = 1);$$

$$X_{6i} = source\ of\ drinking\ water\ associated\ with\ the\ i^{th}\ child\ (piped\ water = 1,\ otherwise = 0);$$

$$X_{7i} = consumption\ of\ \text{“}yam, sweet\ potato, or\ dasheen\text{”}\ associated\ with\ the\ i^{th}\ child\ (No\ consumption = 0;\ otherwise = 1);$$

$$X_{8i} = consumption\ of\ \text{“}carrot\ or\ pumpkin\text{”}\ associated\ with\ the\ i^{th}\ child\ (No\ consumption = 0;\ otherwise = 1);$$

$$X_{9i} = consumption\ of\ \text{“}callaloo, broccoli, or\ pak\ choi\text{”}\ associated\ with\ the\ i^{th}\ child\ (No\ consumption = 0;\ otherwise = 1;$$

$$X_{10i} = consumption\ of\ \text{“}cabbage\text{”}\ associated\ with\ the\ i^{th}\ child\ (No\ consumption = 0;\ otherwise = 1);$$

$$X_{11i} = consumption\ of\ \text{“}avocado\text{”}\ associated\ with\ the\ i^{th}\ child\ (No\ consumption = 0;\ otherwise = 1);$$

$$X_{12i} = frequency\ of\ seafood\ consumption\ associated\ with\ the\ i^{th}\ child = number\ of\ seafood\ meals\ consumed\ in\ a\ week;$$

$$X_{13ij} = \begin{cases} 1, if\ the\ i^{th}\ child\ in\ the\ j^{th}\ matched\ pair\ is\ an\ ASD\ case \\ 0, if\ the\ i^{th}\ child\ in\ the\ j^{th}\ matched\ pair\ is\ a\ control \end{cases};$$

$E(Y_{1i}|\, X_{1i}, X_{13ij})\ stands\ for\ mean\ ln\ of\ blood\ arsenic\ concentrations\ given\ X_{1i}, X_{13ij};$

$E(Y_{2i}|X_{1i}, X_{2i}, \dots, , X_{13ij})\ stands\ for\ mean\ ln\ of\ blood\ arsenic\ concentrations\ given\ the\ X_{1i}, X_{2i}, \dots, X_{12i}, X_{13ij};$

$\beta_{10}, \beta_{11}, \beta_{12}, \beta_{113j}\ are\ regression\ coefficients\ associated\ with\ the\ univaribale\ model;$

$\beta_{20}, \beta_{21}, \dots, \beta_{212}, \beta_{213j}\ are\ regression\ coefficients\ associated\ with\ the\ final\ multivaribale\ model.$

© 2014 by the authors; licensee MDPI, Basel, Switzerland. This article is an open access article distributed under the terms and conditions of the Creative Commons Attribution license (http://creativecommons.org/licenses/by/3.0/).
